# Filamentous bacteriophage M13 induces proinflammatory responses in intestinal epithelial cells

**DOI:** 10.1128/iai.00618-24

**Published:** 2025-04-10

**Authors:** Ambarish C. Varadan, Juris A. Grasis

**Affiliations:** 1 Department of Molecular and Cellular Biology, University of California 33244 , Merced, California, USA; 2 Quantitative and Systems Biology Graduate Group, University of California 33244 , Merced, California, USA; University of Pennsylvania, Philadelphia, Pennsylvania, USA

**Keywords:** enteric virome, bacteriophage, filamentous bacteriophage, inovirus, M13, Fd, intestinal epithelial cells (IECs)

## Abstract

Bacteriophages are the dominant members of the human enteric virome and can shape bacterial communities in the gut; however, our understanding of how they directly impact health and disease is limited. Previous studies have shown that specific bacteriophage populations are expanded in patients with Crohn’s disease (CD) and ulcerative colitis (UC), suggesting that fluctuations in the enteric virome may contribute to intestinal inflammation. Based on these studies, we hypothesized that a high bacteriophage burden directly induces intestinal epithelial responses. We found that filamentous bacteriophages M13 and Fd induced dose-dependent IL-8 expression in the human intestinal epithelial cell line HT-29 to a greater degree than their lytic counterparts, T4 and ϕX174. We also found that M13, but not Fd, reduced bacterial internalization in HT-29 cells. This led us to investigate the mechanism underlying M13-mediated inhibition of bacterial internalization by examining the antiviral and antimicrobial responses in these cells. M13 upregulated type I and III IFN expressions and augmented short-chain fatty acid (SCFA)-mediated LL-37 expression in HT-29 cells. Taken together, our data establish that filamentous bacteriophages directly affect human intestinal epithelial cells. These results provide new insights into the complex interactions between bacteriophages and the intestinal mucosa, which may underlie disease pathogenesis.

## INTRODUCTION

The human enteric virome is composed of eukaryotic and prokaryotic viruses, including viruses that infect human cells, viruses that infect microbes (such as bacteria, fungi, and archaea), and plant viruses that are primarily derived from the environment and diet (1). Alterations in the enteric virome have been reported in colorectal cancer (2, 3), inflammatory bowel disease (4–7), obesity (8), type I diabetes (9), nonalcoholic fatty liver disease (10), cystic fibrosis (11), graft-versus-host disease (12), as well as malnutrition (13). Furthermore, enteric viromes from disease states have been shown to elicit proinflammatory responses, demonstrating their ability to autonomously influence intestinal homeostasis (14). Bacteriophages are the dominant component of the enteric virome (15). These viruses infect bacteria and play a crucial role in shaping bacterial communities in mammalian systems (16). The bulk of the human-associated virome resides in the distal gastrointestinal tract and is composed of tailed double-stranded (ds) DNA bacteriophages (dsDNA phages) (17, 18) that are classified under the class *Caudoviricetes* (19). Metagenomic analyses have reported that patients with ulcerative colitis (UC) have a greater abundance of *Caudovirecetes* bacteriophages (4, 20) and fewer *Microviridae* bacteriophages within their intestines (4), indicating that bacteriophage populations are skewed in these disease states. However, the ability of bacteriophages to directly stimulate human intestinal epithelial cells has not been extensively explored. Although bacteriophages do not directly infect human cells, they do possess molecules that can stimulate the immune system (21) and have been reported to elicit cytokines and antiviral responses in both murine and human leukocytes (22–24). However, it remains unclear whether bacteriophages can directly stimulate the intestinal epithelium and potentially affect disease states in humans.

Gut bacteriophages consist of temperate phages located within bacterial genomes and free lytic bacteriophages associating with the intestinal mucus during the steady state (25). Virulent bacteriophages follow a lytic lifecycle wherein each infection is followed by virion production and host cell lysis (25). The lytic bacteriophages used in this study were T4 and ϕX174. T4 has been shown to induce the expansion of CD4+ and CD8+ T cells in Peyer’s patches of germ-free mice (23). Previous studies have used bacteriophage ϕX174 as a T cell-dependent neoantigen for the assessment of antibody responses in patients (26, 27). Temperate bacteriophages follow a lysogenic lifecycle wherein they integrate into the host bacterial chromosome as a prophage. Prophages are induced upon exposure to specific signals, including antibiotics (28), short-chain fatty acids (SCFAs) (29), reactive oxygen species (30), temperature (31), and food compounds (32), indicating that they re-enter the lytic cycle and cause bacterial lysis and phage release (25). This emergent release of phages could potentially provide a source of antigenic stimuli for intestinal epithelial cells. In addition to lytic and temperate bacteriophages, a recent study reported the detection and characterization of novel inoviruses from gut commensal bacteria (33). Filamentous bacteriophages (or inoviruses) are a subgroup of *Inoviridae*, a family of non-enveloped, single-stranded DNA bacteriophages. They infect both gram-positive and gram-negative bacterial species as well as some species of archaea (34). A unique feature of filamentous bacteriophages is their ability to establish chronic lifecycles, wherein progeny virions are continuously extruded out of the bacterial cell envelope without lysing their host. They adhere to either of two life cycles: episomally replicating phage or temperate phage that can integrate into the host chromosome (35). Filamentous phages have been implicated in bacterial pathogenesis by contributing to biofilm formation (36), promoting bacterial colonization of epithelial cells (37), and increasing the virulence of bacterial wound infections (22). They have also been shown to directly impact mammalian immunity by altering cytokine production in macrophages (22) and chemokine production in keratinocytes (38). The filamentous bacteriophages used in this study were M13 and Fd, which are episomally replicating phages. M13 has been shown to switch the immunosuppressive phenotype of tumor-associated macrophages (TAM) to an inflammatory M1 phenotype (39). Bacteriophage Fd has been shown to stimulate TNF production in bone marrow-derived dendritic cells (BMDCs) (22). Despite the abundance and presence of bacteriophages in the mucosa, very few studies have been conducted on their direct impact on intestinal epithelial cells. In this study, we investigated the mucosal and functional responses of intestinal epithelial HT-29 cells to lytic bacteriophages T4 and ϕX174, and filamentous bacteriophages M13 and Fd. Based on growing evidence for the ability of bacteriophages to interact with mammalian cells (22–24, 36–39), we hypothesized that an increased bacteriophage burden could directly stimulate mucosal responses in intestinal epithelial cells.

## MATERIALS AND METHODS

### Bacterial strains and bacteriophage stocks

The bacteriophage stocks and their respective host bacterial strains used in this study are listed in Table 1. The four *E. coli* strains used in this study were obtained from the Leibniz Institute DSMZ-German Collection of Microorganisms and Cell Cultures GmbH (Leibniz, Germany). The strains were identified as follows: *Escherichia coli* B (DSM 613), *E. coli* PC0886 (DSM 13127), *E. coli* Lederberg (DSM 5695), and *E. coli* LE392 (DSM 4230). Bacteria were cultured on 1% Luria Bertani (LB) (Fisher Scientific) agar (Fisher Scientific) plates and incubated at 37°C overnight. One colony was subsequently used to inoculate a 50  mL tube containing 20  mL LB and incubated again overnight at 37°C. The overnight culture was diluted with LB to an OD_600_ of 0.1 and then incubated at 37°C for 2 h to reach an OD_600_ of 0.5 (corresponding to 10^6^ colony-forming units [CFU/mL]). This was determined to be the optimal bacterial titer for the double agar overlay plaque assay (40) to propagate, as well as to determine the concentration of infectious bacteriophage particles.

**TABLE 1:** Bacteriophages and their respective bacterial strains used in this study

Bacteriophage	Bacteriophage family	Bacterial host
T4 (ATCC 11303-B4)	*Straboviridae*	*E. coli B* (DSM 613)
phiX174 (ATCC 13706-B1)	*Microviridae*	*E. coli* strain PC0886 (DSM 13127)
M13 (DSM 13976)	*Inoviridae*	*E. coli* strain W1485 (DSM 5695)
Fd (DSM 4498)	*Inoviridae*	*E. coli* strain HfrD (DSM 8226)

### Determining bacteriophage titer

Bacteriophage titers were determined using the double agar overlay method (40). Serial dilutions of the bacteriophage stocks were prepared. Phage dilutions (150 µL) were mixed with 150 µL of the respective bacterial host strains (10^8^  CFU/mL). Three milliliters of molten (55°C) 0.5% LB Agar (Fisher Scientific) were added to the phage-bacteria mixture and then plated onto Petri dishes, filled with a bottom layer of 1% LB agar, and incubated for 16  h at 37°C. To determine the stock bacteriophage concentration, plates containing 10 to 200 distinguishable plaques were counted.

### Bacteriophage purification

Bacteriophages were purified using a combination of the Phage-on-Tap protocol (41) and polyethylene glycol-precipitation (42). Bacteria were infected with stocks of bacteriophages at mid-log phase and cultured in 25 mL of LB broth for 16 h at 37°C under shaking conditions. Bacteria were removed by centrifugation at 7,000 × *g* for 30 min, and the supernatant was treated with 1 µg/mL DNase I (Roche) for 2 h at 37°C before 0.22 µm filtration. The virus-containing filtrate was then precipitated with 0.5 M NaCl and 8% polyethylene glycol (PEG) 8000 (Millipore Sigma) overnight at 4°C. The phages were pelleted by centrifugation at 7,000 × *g* for 30 min, and the pellet was suspended in sterile SM buffer (200 mM NaCl_2_, 10 mM MgSO_4_, 50 mM Tris-HCl, pH 7.5). The suspension was centrifuged at 7,000 × *g* for 30 min, and the supernatant was subjected to another round of PEG precipitation. The purified filamentous phage pellets were suspended in sterile SM, incubated with 1-Octanol at 4°C for 2 h to remove endotoxins from the precipitates, dialyzed in a 50 kDa Spectra Por Float-A-Lyzer G2 Dialysis Device (Cole-Parmer) against sterile SM buffer for 48 h, and quantified using double agar overlay assays. Bacteriophage preparations were then tested for endotoxin by *Limulus* amoebocyte lysate (LAL) testing using the Pierce Chromogenic Endotoxin Quantification kit (Thermo Fisher Scientific). Purified bacteriophage preparations were then diluted in SM buffer to working concentrations (1 × 10^9^ PFU/mL) and were tested for endotoxin by Limulus amoebocyte lysate testing. Endotoxin content of the working concentrations of the respective bacteriophage preparations are listed in Table 2.

**TABLE 2:** Titers of bacteriophages and endotoxin levels

Bacteriophage	Titer (PFU/mL)		Working stock endotoxin (10^9^ PFU/mL)			Experimental endotoxin (10^3^ PFU/HT-29)	
	Lysate	Purified	Lysate (EU/mL)	Purified (EU/mL)	Purified (ng/mL)	Purified (EU/mL)	Purified (ng/mL)
T4	1.93 × 10^11^	1.53 × 10^10^	956.00	13.00	1.3	1.3	0.13
phiX174	6.60 × 10^10^	1.73 × 10^10^	1508.58	16.34	1.6	1.6	0.16
M13	1.50 × 10^12^	3.17 × 10^11^	3022.46	50.45	5.0	5.0	0.50
Fd	3.00 × 10^13^	3.23 × 10^11^	1565.20	71.03	7.1	7.1	0.71

### Cell culture

The human intestinal cell line HT-29 was obtained from the Cell Culture Facility at the University of California, Berkeley (Berkeley, CA, USA). HT-29 cells were cultured in Dulbecco’s modified Eagle’s medium (DMEM; Gibco; Thermo Fisher Scientific Inc.) supplemented with 10% (vol/vol) fetal bovine serum (FBS; Gibco; Thermo Fisher Scientific, Inc.), 100 U/mL Penicillin, and 100 µg/mL streptomycin (Gibco; Thermo Fisher Scientific, Inc.). Cells were propagated in a CO_2_ incubator (5% CO_2_) at 37°C and split 1:2 cells to media every 3 days using 0.25% Trypsin-EDTA (Gibco; Thermo Fisher Scientific, Inc). For activation and invasion assays, HT-29 cells were seeded in 12-well tissue culture-treated plates at a concentration of 3.0 × 10^5^ cells per well, and the confluency was determined to be 10^6^ cells per well. Four days after seeding, the confluent cells were serum-starved for 24 h prior to stimulation with bacteriophages. For the kinetics assays, cells were treated with 10 ng/mL phorbol 12-myristate 13-acetate (PMA, Fisher Scientific) and 500 nM Ionomycin (Fisher Scientific) for 3 h, after which they were washed (to remove any residual PMA and Ionomycin) and subsequently treated with purified bacteriophage, LPS, a combination of bacteriophage and LPS, or SM buffer for specified time points. To evaluate antimicrobial peptide (AMP) expression, confluent HT-29 monolayers were treated with 0.5 mM Sodium Butyrate (Fisher Scientific) for 24 h before bacteriophage stimulation, washed, and experimentally treated.

### RNA isolation and cDNA synthesis

The expression levels of selected genes were determined by reverse transcription-quantitative PCR. HT-29 cells treated with PMA/Ionomycin before being mock-stimulated with SM buffer or with purified bacteriophage preparations (10^3^ PFU/HT-29), LPS, or a combination of purified bacteriophage and LPS, for 2, 6, 12, and 24 h at 37°C. Total RNA was extracted from cultured HT-29 cells using TRIzol reagent (Thermo Fisher Scientific) according to the manufacturer’s protocol. The Nanodrop One/One UV-Vis Spectrophotometer (Thermo Fisher Scientific) was used to determine the samples' RNA purity. RNA concentrations were determined using a Promega Quantus Fluorometer (Thermo Fisher Scientific). Equal mass amounts of total RNA were reverse transcribed using Superscript III Reverse Transcriptase (Thermo Fisher Scientific).

### Real-time reverse transcription-quantitative polymerase chain reaction (RT-qPCR)

Real-time RT-qPCR was performed using a StepOnePlus thermocycler (Applied Biosystems). Primers used for RT-qPCR are listed in Table 3. RT-qPCR for each gene was determined in triplicate, and each experiment was repeated at least three times. The final volume of the reaction cocktail was 20 µL, containing 1× PowerUP SYBR Green Master Mix (Thermo Fisher Scientific), 0.5 µM of each primer, and 10 ng of cDNA. The RT-qPCR protocol consisted of one step at 50°C for 2 min (UDG activation) and 95°C for 2 min (initial denaturation), followed by 40 cycles of amplification (95°C for 15 s, 60°C for 30 s, and 72°C for 30 s). Levels of gene expression were normalized to the expression of glyceraldehyde 3-phosphate dehydrogenase (*GAPDH*) control genes. For data analysis, the ΔΔCt method was used to determine the fold change for all target genes in each sample. Data acquisition and analysis were performed using the StepOne Plus Design and Analysis software (version 2.0).

**TABLE 3:** RT-qPCR primers used in this study^*a*^

Gene	Primer sequence (5’−3’)	Product size
GAPDH	Forward (50F): 5′‐CCAGCCGAGCCACATCGCTC‐3′ Reverse (389R): 5′‐ATGAGCCCCAGCCTTCTCCAT‐3′	359 bp
IL-8	Forward (702F): 5′-GGCCAAGAGAATATCCGAACT-3′ Reverse (936R): 5′-GTGAGGTAAGATGGTGGCTAAT-3′	255 bp
TNFα	Forward (994F): 5′-GTCGGAACCCAAGCTTAGAA-3′ Reverse (1247R): 5′-GAAACATCTGGAGAGAGGAAGG-3′	275 bp
IFNα	Forward (37F): 5′-TCAGCAAGCCCAGAAGTATC-3′ Reverse (264R): 5′-GGAACTGGTTGCCATCAAAC-3′	247 bp
IFNβ	Forward (361F): 5′-TAGCACTGGCTGGAATGAG-3′ Reverse (614R): 5′-GTTTCGGAGGTAACCTGTAAG-3′	273 bp
IFNλ	Forward (129F): 5′-CAGCCTCAGAGTGTTTCTTC-3′ Reverse (355R): 5′-GCGACTCTTCTAAGGCATCTT-3′	247 bp
LL-37	Forward (173F): 5′-TGCTAACCTCTACCGCCTCCT-3′ Reverse (289R): 5′-CACAATCCTCTGGTGACTGCT-3′	136 bp
hβD1	Forward (5F): 5′-CTCTGTCAGCTCAGCCTC-3′ Reverse (263R): 5′-CTTGCAGCACTTGGCCTTCCC-3′	278 bp

^*a*^ GAPDH, Glyceraldehyde 3-Phosphate Dehydrogenase; IL-8, interleukin-8; TNFα, tumor necrosis alpha; IFNα, interferon-alpha; IFNβ, interferon-beta; IFNλ, interferon-lambda; LL-37, cathelicidin; hβD1, human beta-defensin 1.

### Viability/cytotoxicity assay

To measure apoptosis, we used the LIVE/DEAD Viability/Cytotoxicity Kit for mammalian cells (Thermo Fisher Scientific), which determines intracellular esterase activity and plasma membrane integrity (43). Briefly, confluent HT-29 monolayers were stimulated either with SM buffer, bacteriophage M13 or Fd, LPS, or a combination of M13 or Fd and LPS for 24 h. HT-29 monolayers were then washed with DMEM (without FBS) and trypsinized with 0.25% trypsin-EDTA for 5 min. After trypsinization, complete cell culture media (DMEM with 10% FBS) was added, and the cell suspensions were centrifuged at 125 × *g* for 5 min. After centrifugation, the cell pellets were stained with LIVE/DEAD Viability/Cytotoxicity Kit for mammalian cells (Thermo Fisher Scientific) by diluting working concentrations of calcein-AM and ethidium homodimer-1 (EthD-1) in Dulbecco’s phosphate-buffered saline (DPBS, Gibco) and adding it to the wells. The final concentrations were 2 µM for calcein-AM and 4 µM for EthD-1. Stained cells were incubated on ice for 10 min. Samples were placed in 12-well plates for quantitative evaluation of viable and dead cell numbers using the LSR II flow cytometer (BD Biosciences). The Calcein-AM signal from viable cells was detected 15 min after dye addition using an excitation filter at 485 nm and an emission filter at 517 nm. The EthD-1 signal from dead cells was detected 15 min after dye addition using an excitation filter at 530 nm and an emission filter at 617 nm. Cells treated with 20% DMSO for 24 h were used as a positive control for EthD-1 staining.

### Cytometric bead assay

To measure the release of IL-8 cytokine, we used the Cytometric Bead Assay (CBA) Human Inflammatory Cytokine Kit (Becton-Dickinson). Briefly, confluent HT-29 monolayers were stimulated either with SM buffer, bacteriophage M13 or Fd, LPS, or a combination of M13 or Fd and LPS for 24 h. The assays were performed according to the manufacturer’s protocol, with the supernatant collected 24 h after HT-29 stimulation. For the CBA kit, 50 µL of supernatant was mixed with the human cytokine capture bead suspension and stained with the PE detection reagent. After 3 h of incubation, the samples were washed and then analyzed using BD CBA software. Human inflammatory cytokine standards provided with the kit were diluted and used in parallel to samples for the preparation of the standard curves.

### Gentamicin protection assay

To determine bacterial internalization by intestinal epithelial cells, gentamicin protection assays were performed as previously described (44, 45), with minor modifications. HT-29 cells were seeded in 12-well tissue culture-treated plates at a concentration of 3.0 × 10^5^ cells per well. Four days after seeding, the cells reached confluency (approximately 10^6^ cells per well) and were serum-starved for 24 h prior to conducting the experiments. Confluent monolayers were washed three times with DMEM (without antibiotics or FBS) and stimulated with SM buffer, 10^3^ PFU/HT-29 M13 or Fd, 100 ng/mL LPS, a combination of 10^3^ PFU/HT-29 M13 or Fd and 100 ng/mL LPS for 6 h. Cells were then washed with DMEM (without antibiotics or FBS) and challenged with overnight-diluted *E. coli* culture (10^7^ CFU/mL) at a multiplicity of infection (MOI) of 10:1 for 6 h. The cell monolayers were washed three times with DMEM (without antibiotics or FBS) prior to treatment with 100 µg/mL gentamicin sulfate (Fisher Scientific) for 1 h. The cells were then lysed using 0.1% Triton X-100 lysis buffer (Fisher Scientific). Serial dilutions of the lysates were plated on LB agar, and bacterial colonies were counted after 16 h of incubation at 37°C.

### Statistical analyses

All experiments were conducted at least three times. Individual data points are displayed when possible and are represented as the mean  ±  standard error of the mean (±SEM). Statistical significance was calculated using GraphPad PRISM software (version 10 for Windows; GraphPad Software, Inc.). The Shapiro-Wilk test was used to determine whether the data were normally distributed. Statistical significance was calculated using a two-tailed Student’s *t*-test or Analysis of Variance (ANOVA) with Tukey’s or Dunnett’s multiple comparison correction, where two or more groups were compared. *P*  <  0.05 was considered statistically significant.

## RESULTS

### Bacteriophages induce proinflammatory cytokine activation in epithelial cells

**Fig 1:**
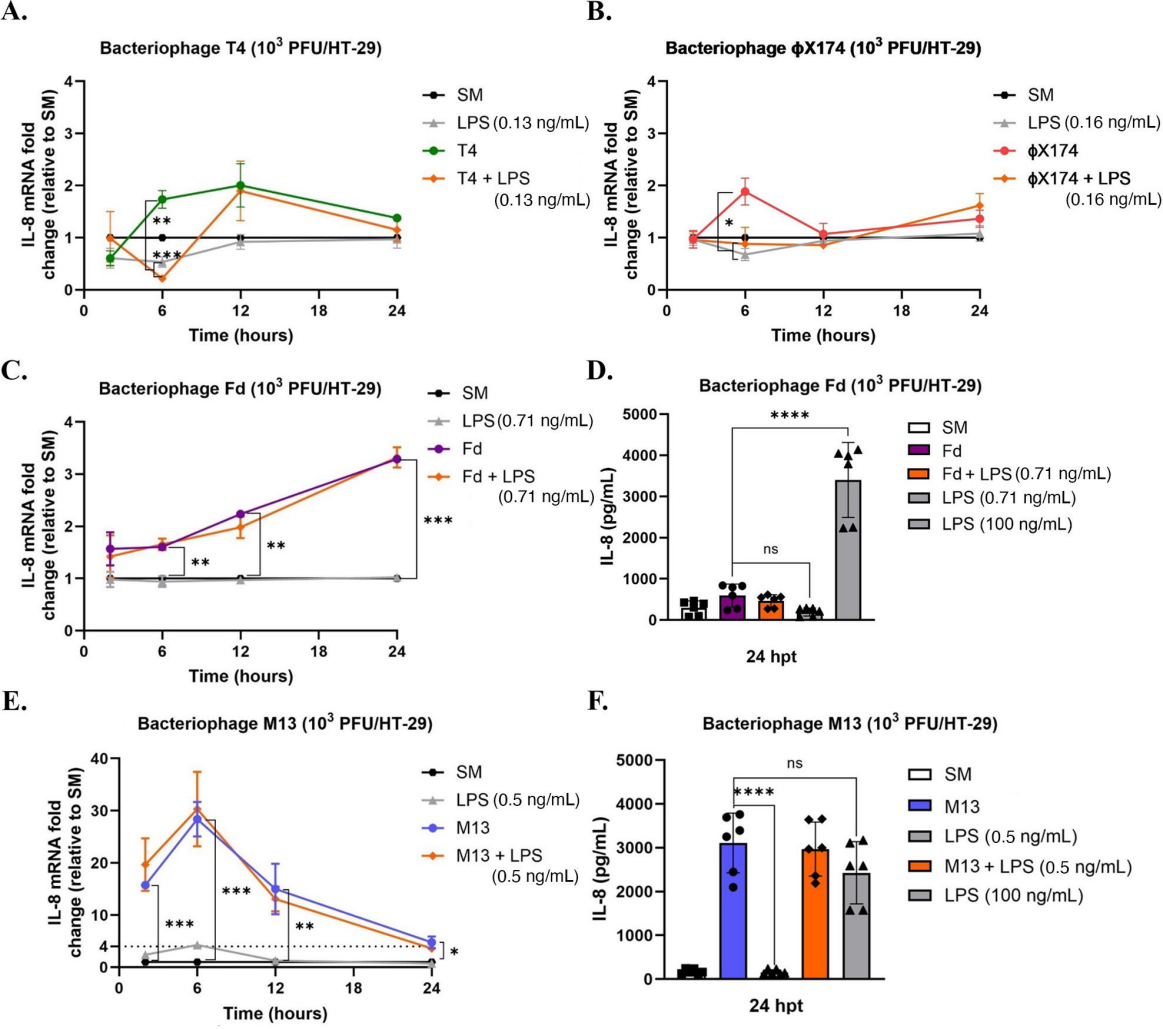
Kinetics of bacteriophage-mediated IL-8 activation of HT-29 epithelial cells. Figure 1. Kinetics of bacteriophage-mediated IL-8 activation. Primed HT-29 cells were stimulated with 10^3^ PFU/HT-29 bacteriophages (A) T4, (B) ϕX174, (C) Fd, and (D) M13 for 2, 6, 12, and 24 h. Respective controls for each experiment were SM buffer, LPS, bacteriophage, and a combination of bacteriophage with LPS. Graphs (A) to (D) are representative of *n* ≥ 3 experiments and depict the mean with ±SEM of *n* ≥ 3 replicates from an individual experiment, each dot indicating the replicate value. The comparative 2^-ΔΔCt^ method was used to quantify gene expression level changes in the respective controls relative to SM buffer after normalization to the housekeeping gene GAPDH. Non-primed HT-29 cells were stimulated with 10^3^ PFU/HT-29 (E) M13 and (F) Fd for 24 h prior to evaluating IL-8 secretion through a cytometric bead assay. Respective controls for each experiment were SM buffer, bacteriophage, LPS (at levels present in the bacteriophage preparation), a combination of bacteriophage with LPS, and a high concentration of LPS to serve as a positive control. Graphs (E) and (F) are representative of *n* = 3 experiments and depict the mean with ±SEM of *n* = 6 replicates; each dot indicates the replicate value. Analysis: one-way ANOVA with Tukey’s test for multiple comparisons. * *P* < .05, ** *P* < .01, *** *P* < .001, **** *P* < .0001.

We assessed the immunogenicity of lytic bacteriophages T4 and ϕX174, and filamentous bacteriophages M13 and Fd in the colonic epithelial cell line HT-29, which is widely used to model the immune function of intestinal epithelial cells (46, 47). We hypothesized that bacteriophages increase the expression of the cytokine IL-8 in intestinal epithelial HT-29 cells, a proinflammatory marker for these cells. Our rationale for targeting IL-8 expression is that it is a major human chemokine that is rapidly synthesized in large amounts by intestinal epithelial cells at both mRNA and protein levels (48, 49). One challenge in studying mammalian immune responses to bacteriophages is the removal of endotoxins from the bacteriophage lysates. Bacterial endotoxins are highly immunogenic and can trigger inflammatory responses in TLR4-expressing mammalian cells (50, 51). We purified all bacteriophage lysates of endotoxin through a combination of the Phage-on-Tap (41) and the polyethylene glycol precipitation (42) protocols (the titers of working stocks of purified bacteriophage preparations and their respective endotoxin levels are listed in Table 2). We chose LPS from *E. coli* O111:B4 as a control for our cellular activation experiments control since the bacteriophages used *E. coli* as a host in the experiments. Previous studies have shown that intestinal epithelial cells are hyporesponsive to extracellular LPS (52–55) due to diminished TLR4 expression (53, 55) and the lack of coreceptor MD-2^53^. In HT-29 cells, TLR4 protein is largely present in the cytoplasmic fraction, and the cells are hyporesponsive to LPS in an unprimed condition (55). Therefore, we primed the cells with PMA/Ionomycin (56) prior to bacteriophage treatment. Following previous studies that have examined bacteriophage immunogenicity utilizing ratios ranging from 10^1^ to 10^4^ PFU bacteriophage/mammalian cell (22, 24, 38, 57, 58), HT-29 cells were stimulated with 10^1^–10^3^ PFU bacteriophage/HT-29, depending upon the assay. To determine whether the observed immune response was induced by the bacteriophage rather than by possible endotoxin contamination present in the purified bacteriophage preparation, we stimulated primed HT-29 cells with exogenous LPS and compared LPS-induced IL-8 expression to bacteriophage-induced IL-8 expression. To determine the immunogenicity of lytic bacteriophage T4, primed HT-29 cells were stimulated with either 10^3^ PFU/HT-29 purified T4, 0.13 ng mL^−1^ LPS, a combination of purified bacteriophage (at a concentration of 10^3^ PFU/HT-29) and 0.13 ng mL^−1^ exogenous LPS, or mock-stimulated with SM buffer for 2, 6, 12, and 24 h prior to evaluating IL-8 expression. As shown in Fig. 1A, bacteriophage T4 induced significantly higher IL-8 expression compared with that induced by LPS, the combination of T4 and LPS, and SM buffer at 6 h. No significant upregulation of IL-8 expression was observed in response to T4 treatment at any of the other time points. The addition of exogenous LPS to 10^3^ PFU/HT-29 bacteriophage T4 also did not lead to a significant difference in IL-8 expression induced by the phage alone at any of these time points. Interestingly, T4 reduced LPS-induced IL-8 activation at 6 h.

To assess the kinetics of lytic bacteriophage ϕX174-mediated HT-29 activation, primed HT-29 cells were stimulated with either 10^3^ PFU/HT-29 of bacteriophage ϕX174, 0.16 ng.mL^−1^ LPS, a combination of purified bacteriophage (at a concentration of 10^3^ PFU/HT-29) and 0.16 ng.mL^−1^ exogenous LPS, or mock-stimulated with SM buffer for the same time(s) as indicated in Fig. 1A. Similar to T4, we observed the highest expression of IL-8 in response to stimulation with bacteriophage ϕX174 at 6 h. We also observed that at 6 h, ϕX174 induced significantly higher IL-8 expression than that induced by the combination of ϕX174 and LPS (Fig. 1B), indicating that lytic bacteriophages can counteract LPS-induced IL-8 expression at specific time points. To assess whether these bacteriophages could activate another proinflammatory cytokine, we evaluated TNFα expression at 6 h. Both T4 and ϕX174 induced significantly higher TNFα expression at 6 h compared with that induced by LPS or the combination of bacteriophage and LPS, although T4 induced greater TNFα expression than ϕX174 (Fig. S1A and B).

Next, we determined the immunogenicity of filamentous bacteriophages by assessing the kinetics of IL-8 expression. HT-29 cells were stimulated with either 10^3^ PFU/HT-29 of purified filamentous bacteriophage Fd (at a concentration of 10^3^ PFU/HT-29), 0.71 ng/mL LPS, a combination of bacteriophage Fd and LPS, or SM buffer for 2, 6, 12, and 24 h (Fig. 1C). At 6, 12, and 24 h post-treatment, bacteriophage Fd induced significantly higher IL-8 expression compared with that induced by LPS and SM buffer, with the highest IL-8 induction observed at 24 h. The addition of exogenous LPS to bacteriophage Fd did not lead to a significant difference in IL-8 expression as induced by the phage alone at any of the time points. Fd did not significantly stimulate IL-8 secretion in HT-29 cells at 24 hours, as assessed by a cytometric bead assay (Fig. 1D). Additionally, Fd did not upregulate TNFα expression at 6 h post-treatment (Fig. S1D). To determine whether priming HT-29 cells with PMA/Ionomycin impacted bacteriophage-mediated stimulation, we also examined whether filamentous bacteriophages could stimulate non-primed HT-29 cells. Bacteriophage Fd did not stimulate significantly higher IL-8 expression than exogenous 0.71 ng/mL LPS in non-primed cells (Fig. S2C). Finally, we determined whether Fd-mediated IL-8 induction occurred in a concentration-dependent manner 24 h post-treatment and found that lowering Fd concentrations significantly reduced IL-8 expression (Fig. S3A).

To assess the effects of filamentous bacteriophage M13-mediated IL-8 activation, HT-29 cells were stimulated with 10^3^ PFU/HT-29 of bacteriophage M13, 0.5 ng/mL LPS, a combination of bacteriophage M13 and LPS, or SM buffer for 2, 6, 12, and 24 h (Fig. 1E). M13 induced significantly higher IL-8 expression compared with that induced by LPS and SM buffer at all evaluated time points, with the highest IL-8 induction observed at 6 h (Fig. 1E). The addition of 0.50 ng/mL exogenous LPS to 10^3^ PFU/HT-29 of bacteriophage M13 did not lead to a significant difference in IL-8 expression induced by the phage alone at any of the time points. M13 also induced greater IL-8 activation than T4, ϕX174, or Fd (the dotted line in Fig. 1E indicates the maximal IL-8 activation observed in response to other bacteriophages). We also evaluated whether M13 stimulated IL-8 expression in non-primed HT-29 cells at 24 h. As shown in Fig. S2B, M13 (10^3^ PFU/HT-29) induced significantly higher IL-8 expression than 0.5 ng/mL LPS or SM buffer at 24 h. The addition of exogenous 0.5 ng/mL LPS to bacteriophage M13 did not lead to a significant increase in IL-8 expression. LPS (100 ng/mL) was used as a positive control (59) and stimulated IL-8 expression greater than M13. We found that M13 induced significantly higher IL-8 secretion compared with 0.5 ng/mL LPS. Furthermore, M13-mediated IL-8 secretion was comparable with that induced by the positive control, 100 ng/mL LPS (Fig. 1E). In addition to IL-8, M13 induced significantly higher proinflammatory TNFα expression than LPS at 6 h post-treatment (Fig. S1C). The addition of exogenous LPS to bacteriophage M13 did not lead to a significant increase in TNFα expression. To determine whether the observed increase in IL-8 and TNFα expression was due to cellular apoptosis of HT-29 cells (60, 61), we evaluated cellular viability in response to filamentous bacteriophage stimulation at 24 h. As shown in Fig. S2A, stimulating HT-29 cells with 10^3^ PFU/HT-29 of purified bacteriophage M13 or Fd did not significantly decrease cellular viability compared with SM buffer-treated HT-29 cells. Next, we determined whether M13-mediated IL-8 induction occurred in a concentration-dependent manner 24 h post-treatment (Fig. S3B). Lowering the bacteriophage M13 concentration from 10^4^ PFU/HT-29 to 10^2^ PFU/HT-29 did not significantly reduce IL-8 expression. Only when the bacteriophage concentration was reduced to 1 PFU/HT-29 and 0.1 PFU/HT-29 did we observe a significant reduction in IL-8 activation.

Collectively, these results suggest that bacteriophages can directly stimulate HT-29 cells with filamentous bacteriophage M13 inducing much higher IL-8 at the transcript and protein levels compared with the other bacteriophages tested. Given that filamentous bacteriophages induced a greater proinflammatory response compared with their lytic counterparts, we focused on intestinal epithelial responses to filamentous bacteriophages M13 and Fd for the rest of this study.

### Stimulation of gut epithelial cells with filamentous bacteriophage M13 reduces bacterial internalization

**Fig 2:**
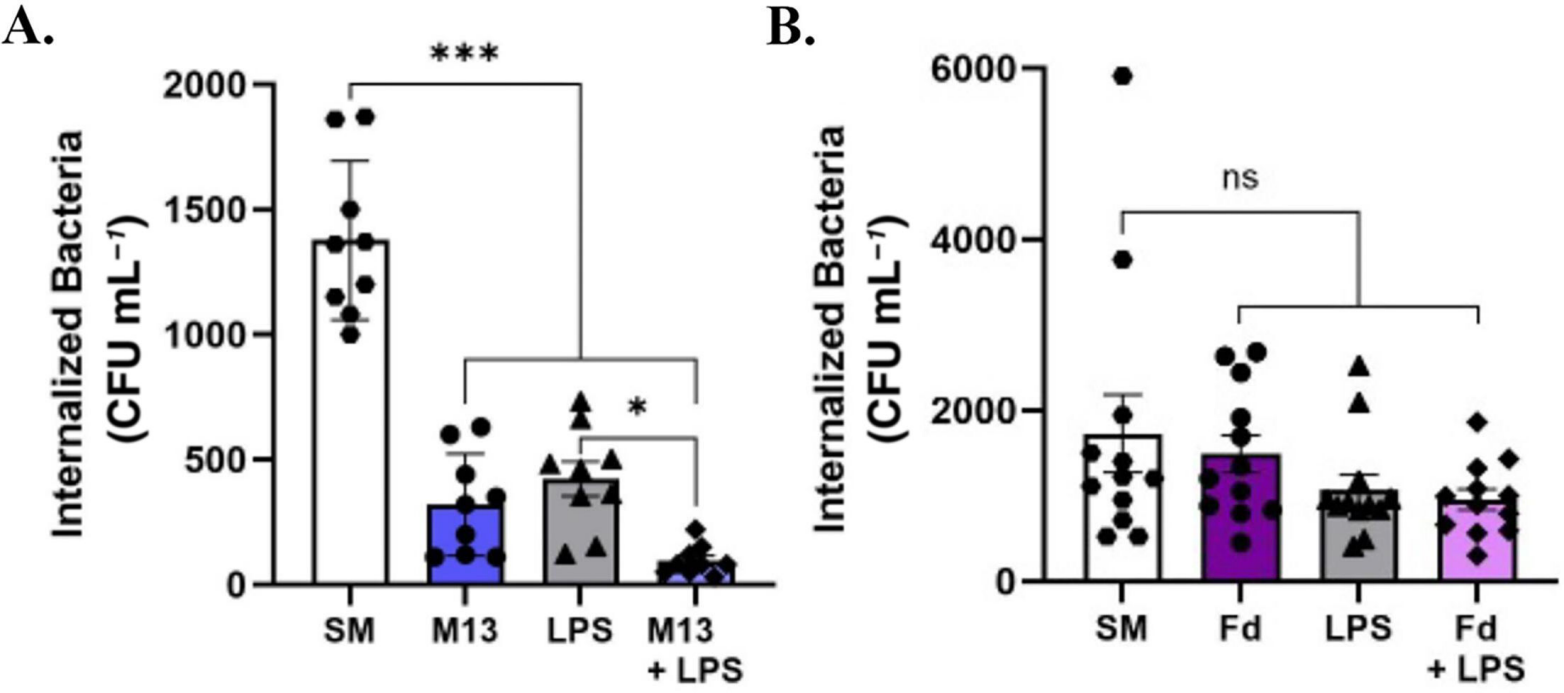
Filamentous bacteriophage M13 reduces bacterial internalization in gut epithelial cells. Figure 2. Bacterial internalization in HT-29 cells is affected by M13. (A) Confluent HT-29 cells (10^6^) were co-incubated with 10^7^ CFU/mL *E. coli* strain W1485 or M13 (10^7^ PFU/mL) and *E. coli* concurrently (phage-bacteria ratio or MOI = 1) for 6 h prior to determining bacterial internalization. (B) Confluent HT-29 cells (10^6^) were co-incubated with 10^7^ CFU/mL *E. coli* strain HfrD or Fd (10^7^ PFU/mL) and *E. coli* concurrently (phage-bacteria ratio or MOI = 1) for 6 h prior to determining bacterial internalization. All graphs are representative of *n* = 3 experiments and depict the mean with ±SEM of *n* = 9 replicates, each dot indicating the replicate value. Statistical analysis was computed based on the nine replicates. Analysis: (A) Two-tailed Student’s *t*-test; (B) one-way ANOVA with Dunnett’s test for multiple comparisons. * *P* < .05, ** *P* < .01, *** *P* < .001.

We next investigated whether filamentous bacteriophages could affect intestinal epithelial internalization and, therefore, affect bacterial infection rate. Bille et al. showed that the presence of filamentous bacteriophages results in increased bacterial colonization of epithelial cells (37), implying that they can play pathogenic roles in bacterial infections of human cells. We hypothesized that the presence of filamentous bacteriophages would increase the number of bacteria that could be internalized by HT-29 cells. To test this, we incubated HT-29 cells with *E. coli* strain W1485 (host of M13) and M13 prior to measuring bacterial internalization. We found that the presence of bacteriophage M13 significantly reduced the number of *E. coli* W1485 cells internalized by HT-29 cells (Fig. S4A). LPS has been shown to increase the permeability of intestinal epithelial tight junctions (62). When HT-29 cells were pre-stimulated with M13 and LPS prior to bacterial infection, we observed reduced bacterial internalization compared with HT-29 cells that were pre-stimulated with LPS prior to infection (Fig. 2A). To determine whether M13 directly acts on HT-29 cells to inhibit bacterial internalization, we stimulated HT-29 cells with M13 prior to infection with *E. coli* 1485. We found that HT-29 cells stimulated with M13 internalized fewer *E. coli* 1485 cells than HT-29 cells stimulated with an equivalent volume of SM buffer (Fig. 2A). Given that M13-mediated activation of HT-29 cells occurred in a concentration-dependent manner and was maintained over time (Fig. S3B; Fig. 1E), we hypothesized that the M13-mediated reduction in bacterial internalization would also occur in a concentration-dependent manner. However, we observed that stimulating HT-29 cells with different concentrations of M13 (10^0^–10^3^ PFU/HT-29) prior to bacterial infection did not significantly alter the number of internalized *E. coli* 1485 cells (Fig. S4B). When HT-29 cells were pre-stimulated with filamentous bacteriophage Fd before infection with *E. coli* strain HfrD (host of Fd), we did not observe a decrease in bacterial internalization compared with HT-29 cells that were pre-stimulated with an equivalent volume of SM buffer prior to infection (Fig. 2B). Co-stimulation with Fd and LPS did not result in reduced internalization of *E. coli* HfrD compared with LPS stimulation alone. It should be noted that W1485 (host of M13) and HfrD (host of Fd) are different strains of the same bacterium *E. coli*. Prestimulation with LPS alone did not impact *E. coli* HfrD internalization (Fig. 2B), but it did impact *E. coli* W1485 internalization (Fig. 2A) by HT-29 cells, suggesting that bacterial internalization in HT-29 cells is strain-dependent. Our experiments demonstrated that M13-mediated reduction in bacterial internalization was not universal across all filamentous bacteriophages.

### Stimulation with bacteriophage M13 triggers antiviral type I and III interferon responses

**Fig 3:**
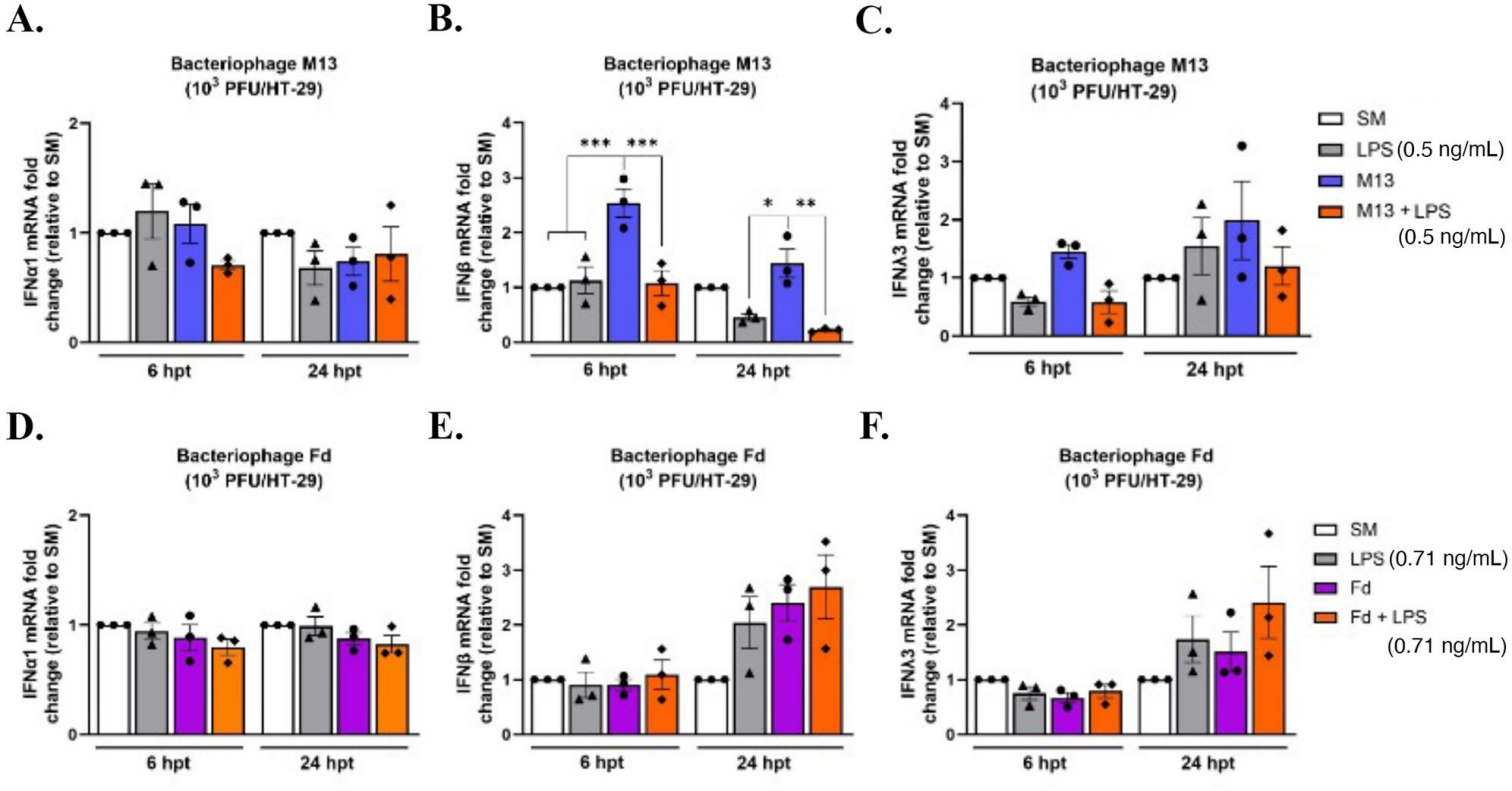
Filamentous bacteriophage M13 triggers antiviral type I and type III interferon responses. Figure 3. M13 triggers antiviral type I and III IFN induction. Effects of bacteriophage M13 stimulation on (A) IFNα, (B) IFNβ, and (C) IFNλ induction at 6 and 24 h, respectively. Effects of bacteriophage Fd stimulation on (D) IFNα, (E) IFNβ, and (F) IFNλ induction at 6 and 24 h, respectively. Respective controls for each experiment were LPS, bacteriophage, and a combination of bacteriophage with LPS. The comparative 2^-ΔΔCt^ method was used to quantify gene expression level changes in the respective controls relative to SM buffer after normalization to the housekeeping gene GAPDH. All graphs are representative of *n* ≥ 3 experiments and depict the mean with ±SEM of *n* ≥ 3 replicates from an individual experiment: each dot indicating the mean experiment value. Analysis: one-way ANOVA with Tukey’s test for multiple comparisons. * *P* < .05, ** *P* < .01, *** *P* < .001.

Next, we sought to determine whether filamentous bacteriophages could stimulate antiviral responses in HT-29 cells. Intestinal epithelial cells play a crucial role in maintaining intestinal homeostasis and regulating microbial colonization through a variety of mechanisms, including antiviral (63), antimicrobial (64), and mucosal (65) responses. Interferons (IFNs) are the main cytokines produced by intestinal cells, which help control viral replication and spread within the body. The human intestinal epithelium exploits two types of IFNs for its protection: type I (IFN-α and IFN-β) and type III IFNs (IFN-λ1, -2, -3, and -4) (63). Type I IFN signaling has also been shown to exert protective effects against bacterial infection (66, 67). Given that filamentous bacteriophages have been shown to promote the production of type I interferon (IFN) in murine BMDCs (22), we hypothesized that they would induce type I and III IFN responses in colonic epithelial HT-29 cells. As shown in Fig. 3A, no significant change in IFNα expression was observed in HT-29 cells stimulated with M13, LPS, or the combination of LPS and M13 at 6 and 24 h. However, bacteriophage M13 significantly induced higher IFNβ expression than LPS, as well as the combination of LPS and M13 at 6 and 24 h (Fig. 3B). Bacteriophage M13 also induced significantly higher IFNλ expression than LPS, as well as the combination of LPS and M13 at 6 h (Fig. 3C). However, no significant change in IFNλ expression was observed in HT-29 cells stimulated with M13, LPS, or the combination of LPS and M13 at 24 h (Fig. 3C). No significant change in IFNα, IFNβ, and IFNλ expression was observed in HT-29 cells stimulated with either Fd, LPS, or the combination of LPS and Fd at either 6 or 24 h (Fig. 3D through F), demonstrating that filamentous bacteriophages exert differential antiviral responses in intestinal epithelial cells.

### Filamentous bacteriophage M13 augments butyrate-mediated LL-37 antimicrobial peptide expression

**Fig 4:**
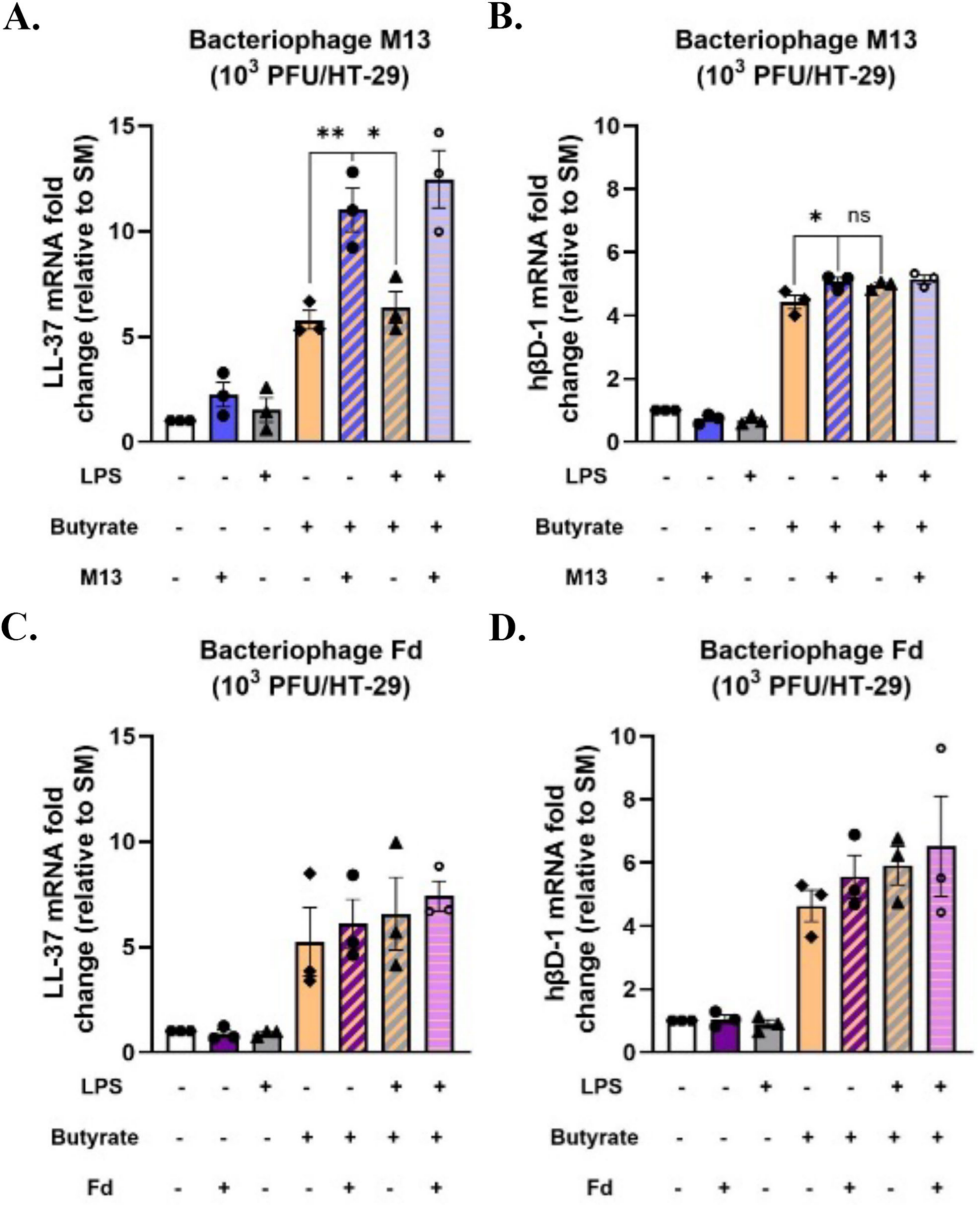
Filamentous bacteriophage M13 augments butyrate-mediated LL-37 antimicrobial peptide expression. Figure 4. M13 augments SCFA butyrate-mediated LL-37 expression. LL-37 expression was assessed in response to bacteriophages (A) M13 and (C) Fd. hBD1 expression was evaluated in response to bacteriophages (B) M13 and (D) Fd. For each experiment, HT-29 cells were stimulated with SM buffer, bacteriophage, LPS, butyrate, or their combinations for 24 h. The comparative 2^-ΔΔCt^ method was used to quantify gene expression level changes in the respective controls relative to SM buffer after normalization to the housekeeping gene GAPDH. All graphs are representative of *n* ≥ 3 experiments and depict the mean with SEM of *n* ≥ 3 replicates from an individual experiment: each dot indicating the replicate value. Analysis: one-way ANOVA with Tukey’s test for multiple comparisons. * *P* < .05, ** *P* < .01.

Based on a previous study that reported that pretreatment of mucus-producing intestinal epithelial cells with bacteriophages reduced subsequent bacterial attachment and cell death (68), we hypothesized that filamentous bacteriophages M13- and Fd-induced antimicrobial peptide (AMP) gene expression in HT-29 cells. Antimicrobial peptides (AMPs) are small (2–5 kDa), cationic, amphipathic peptides that play a critical role in innate immune defense mechanisms (69) against a broad range of microorganisms, including bacteria, fungi, parasites, and viruses (64). We determined whether bacteriophages induced the expression of cathelicidin LL-37 and β-defensin-1 (hβD-1) in HT-29 cells. LL-37 is a small, linear peptide that possesses broad bactericidal activity against both gram-negative and gram-positive bacteria (70). Defensins are small, cationic peptides that contain disulfide bonds that are necessary to damage the bacterial cell membrane and eradicate bacteria (71). β-Defensin hβD-1 is constitutively expressed in the gastrointestinal tract (72). We found that neither M13 (Fig. 4A and B) nor Fd (Fig. 4C and D) significantly upregulated LL-37 or hβD-1 expression compared with buffer-treated HT-29 cells at 24 h post-treatment. Short-chain fatty acids (SCFAs) have been reported to be strong inducers of LL-37 expression in colonocytes (73, 74). They are microbial metabolites that constitute the major products of bacterial fermentation of dietary fiber in the intestines (75). The major SCFAs produced in the colon are acetate, propionate, and butyrate (76). We then investigated whether filamentous bacteriophages would affect AMP gene expression in the presence of butyrate. In agreement with previous studies (73, 74), the administration of butyrate alone significantly upregulated both LL-37 and hβD-1 expressions in HT-29 cells. However, the administration of M13 along with butyrate induced a significantly higher LL-37 expression (Fig. 4A), but not hβD-1 expression (Fig. 4B), compared with that elicited by butyrate alone. The administration of exogenous LPS along with butyrate induced a similar expression of LL-37 as that induced by butyrate alone, confirming that the increased LL-37 expression in response to a combination of bacteriophage M13 and butyrate was not due to any residual LPS present in the bacteriophage preparation. The combination of Fd with butyrate did not significantly upregulate either LL-37 (Fig. 4C) or hβD-1 expression (Fig. 4D) compared with that elicited by butyrate alone. Collectively, these results suggest that specific bacteriophages can synergize with gut metabolites to induce AMP gene expression.

## DISCUSSION

Epithelial cells are the first line of intestinal defense and act as the interface between the intestinal microbiota and the body’s internal milieu (77). Bacteriophages are the most abundant viruses in the gut commensal microbiota, and their abundance has been reported to be altered in many inflammatory disease states (2, 3, 8–13), including IBD (4–7). Although previous studies have shown that bacteriophages can directly stimulate both human and murine phagocytes (22–24), very few studies have been conducted to determine the direct impact of bacteriophages on human intestinal epithelial cells, which provide frontline responses to gut microbiota to maintain intestinal homeostasis. Understanding whether and how bacteriophages evoke intestinal epithelial cellular responses may be the first step in elucidating their potential roles in disease pathogenesis. In this study, we established that bacteriophages can directly stimulate proinflammatory responses in intestinal epithelial cells. These responses were dose-dependent, as in the case of filamentous bacteriophage M13- and Fd-mediated IL-8 activation, and were greater than those induced by their lytic counterparts T4 and ϕX174. Previous studies have reported that bacteriophages can elicit both proinflammatory (78) and anti-inflammatory responses (24) in human cells, implying that their dynamics within the human host are both phage- and cell-specific. We observed differential dynamics between lytic and filamentous bacteriophages with respect to their synergy with lipopolysaccharides (LPS). Lytic (T4 and ϕX174), but not filamentous (M13 and Fd) bacteriophages, decreased the inflammatory response to LPS at 6 h post-treatment, as assessed by the decreased intestinal epithelial expression of IL-8 and TNFα. Miernikiewicz et al. previously reported that T4 short-tail fiber adhesin gp12 decreased LPS-induced proinflammatory cytokines IL-1α and IL-6 *in vivo* (79). Furthermore, Zhang et al. reported that *Staphylococcus aureus* lytic bacteriophages suppressed LPS-induced inflammation in bovine mammary epithelial cells (80). Based on these studies, we hypothesize that lytic bacteriophages may modulate the immunogenicity of LPS through a physical interaction. LPS is a component of the outer membrane of gram-negative *E. coli* and is one of the receptors for both bacteriophages T4 (81) and ϕX174 (82). The first step in phage infection is adsorption to the bacterial cell surface, which involves irreversible binding of T4 to LPS (81). The mechanism(s) underlying bacteriophage modulation of LPS-induced proinflammatory immune responses need to be investigated further and may provide new insights into the development of bacteriophages as a therapeutic option to combat antibiotic-resistant infections. We observed that filamentous bacteriophage M13 induced much higher IL-8 activation than the other phages. Furthermore, M13-mediated IL-8 upregulation was independent of priming the cells, as we observed similar levels of IL-8 upregulation in the presence or absence of PMA/Ionomycin. We also observed M13-induced IL-8 secretion in non-primed HT-29 cells. However, Fd did not significantly upregulate either IL-8 expression or secretion in non-primed HT-29 cells. IL-8, a powerful chemoattractant released by intestinal epithelial cells, attracts neutrophils to the basolateral surface of the epithelium (48). Elevated IL-8 expression has been reported in the inflamed mucosa of patients with ulcerative colitis (83–85). Determining the molecular pathways underlying filamentous bacteriophage-mediated IL-8 activation and whether this activation could increase neutrophil recruitment and inflammation will provide new insights into bacteriophage-induced immune responses and present an avenue for future research.

Previous studies have directly implicated filamentous bacteriophages in the bacterial pathogenesis of human cells. Sweere et al. (22) reported that filamentous bacteriophage Pf4 impaired the clearance of *P. aeruginosa* by both murine and human phagocytes, whereas Bille et al. showed that the presence of filamentous bacteriophage MDAϕ resulted in increased colonization of *Neisseria meningitidis* on epithelial cells (37). Here, we report that M13 reduces the internalization of *E. coli* W1485 in HT-29 cells. In contrast to the above-mentioned studies that used pathogenic bacteria such as *P. aeruginosa* and *N. meningitidis*, the bacterial strains of *E. coli* that we used for the internalization experiments were commensal. Common gut commensal species, such as *E. coli* (86), can be internalized by enterocytes, although in significantly smaller numbers than invasive enteric pathogens (such as *Salmonella typhimurium* and *Listeria monocytogenes*) (87–89). Here, we assessed the invasiveness of *E. coli* W1485 by observing its internalization by HT-29 cells. Although our data demonstrated that pre-stimulation of HT-29 cells with M13 reduced the internalization of *E. coli* W1485, we cannot conclude that M13 protects HT-29 cells against bacterial invasion. Subsequent internalization studies should be conducted with enteric pathogens to evaluate the protective potential of M13. If further studies show that M13 can indeed protect HT-29 cells against pathogenic bacterial invasion, a mechanism of M13-mediated reduction of bacterial internalization could be determined. In the study by Sweere et al. (22), it was demonstrated that monoclonal antibodies generated against the Pf4 major capsid protein CoaB reduced the incidence of *P. aeruginosa* wound infections in addition to promoting the phagocytic engulfment of PAO1 (a strain of *P. aeruginosa* carrying Pf4). Therefore, antibody-mediated recognition of filamentous bacteriophage Pf4 promotes *P. aeruginosa* phagocytosis (22). Given that the major capsid protein pVIII forms the body of bacteriophage M13 and is the most abundant protein on the surface of the bacteriophage virion (35), it would be interesting to evaluate whether targeting the major capsid protein of M13 would affect bacterial internalization by HT-29 cells. We hypothesize that antibody-mediated recognition of pVIII would increase bacterial internalization by HT-29 cells. Very few studies have examined the immunogenicity of individual bacteriophage proteins (79, 90). Results from these proposed experiments would indicate whether the major capsid protein pVIII of bacteriophage M13 is actively involved in modulating intestinal immunity. We did not observe a significant reduction in bacterial internalization by HT-29 cells when they were pre-stimulated with filamentous bacteriophage Fd, suggesting that M13 and Fd may employ potentially differential interactions with colonic epithelial cells. The reason(s) behind this differential interaction is unclear and needs to be investigated further, but it may be related to differences in their respective capsid protein structure or amino acid composition.

These differences may also have affected the differences in endotoxin between the filamentous and lytic phages (Table 2). These differences may be due to the significant size and structure differences of the filamentous phages as compared with the lytic phages. The levels of endotoxin in the filamentous phage preps, although significantly reduced, are at or slightly above clinically approved levels. We spent considerable time and resources to reduce the levels of endotoxin in the filamentous phage preps but unfortunately could not reduce the endotoxin levels further without significant loss of phage. These differences were addressed experimentally by using different amounts of LPS as controls equivalent to the phage preps. Since these LPS controls did not affect results, we can be confident in our phage-mediated conclusions.

We observed that M13 induced antiviral type I and type III IFN expressions in HT-29 cells, corroborating previous studies that demonstrated that filamentous bacteriophages can trigger type I IFN production in phagocytes (22). Recently, single-cell RNA sequencing revealed that filamentous bacteriophages upregulate several antiviral response genes in human basal epithelial cells (BCs), including IRF7^58^, a key transcription factor downstream of TLR3/TRIF signaling, primarily induced by type I IFNs and viral sensing (91). Given that type I IFN signaling has also been shown to have protective effects against bacterial infection (66, 67), it is likely that M13-mediated type I IFN induction could play a role in regulating bacterial internalization by HT-29 cells. Alternatively, Sweere et al. demonstrated that filamentous bacteriophage Pf4 stimulated TLR3- and TRIF-dependent type I IFN production, inhibited TNF production, and limited phagocyte-mediated clearance of *P. aeruginosa* (22). This is an example of a filamentous bacteriophage-mediated maladaptive antiviral response that results in impaired bacterial clearance and an increased establishment of infection. Conducting a global transcriptomic analysis of filamentous bacteriophage-stimulated intestinal epithelial cells will be informative, not only in determining which antiviral response genes are induced but also in providing insight(s) into the sensing mechanisms used by these cells to recognize filamentous bacteriophages. This could be followed by functional studies to evaluate whether M13 could reduce bacterial internalization in intestinal epithelial cells lacking key components of the antiviral induction pathway. This would confirm whether M13-induced antiviral responses are protective or pathogenic in the context of bacterial infections of intestinal epithelial cells.

The key to delineating the mechanism by which M13 inhibits bacterial internalization by colonic epithelial cells may lie in the components of the mucosal surface. MUC2 is the main macromolecular component of intestinal mucus (92), and previous reports have demonstrated that bacteriophages can adhere to (68) and persist within mucosal surfaces (93). Furthermore, Le et al. showed that the mucus layer in colonoid-derived monolayers prevented bacteriophage translocation, demonstrating the importance of colonic mucus in preventing bacteriophage translocation (94). This would suggest that M13 might interact with and infect its host bacteria at the mucosal surface, thereby regulating bacterial internalization by intestinal epithelial cells through a mucus-dependent mechanism. Future studies aimed at depleting the mucus layer in HT-29 cells prior to evaluating bacterial internalization would confirm whether M13-mediated reduction in bacterial internalization is mucus-dependent. Alternatively, Tian et al. reported that M13 can enter epithelial cells through clathrin-mediated endocytosis and macropinocytosis (95). Given that both phages (22, 96) and commensal bacteria have been shown to be internalized by human cells, it is also likely that internalized M13 could regulate bacterial populations intracellularly (97) through a mucus-independent mechanism.

In conclusion, these studies established that bacteriophages have direct effects on human intestinal epithelial cells and suggested that bacteriophages may play crucial roles in bacterial infections by directly interacting with intestinal epithelial cells.

## Data Availability

The data supporting the findings of this study are available within the article and/or its supplemental material.

## SUPPLEMENTAL MATERIAL

Supplemental figures iai.00618-24-s0001.docx Fig. S1 to S4.

ASM does not own the copyrights to Supplemental Material that may be linked to, or accessed through, an article. The authors have granted ASM a non-exclusive, world-wide license to publish the Supplemental Material files. Please contact the corresponding author directly for reuse.
