# Supplementary material for: Filamentous bacteriophage M13 induces proinflammatory responses in intestinal epithelial cells
Source: Infect Immun. 2025 Apr 10;93(5):e00618-24. doi: 10.1128/iai.00618-24 (PMC12070739; doi:10.1128/iai.00618-24)
Supplement: Supplemental figures — Fig. S1 to S4. [file iai.00618-24-s0001.docx]

**Supplementary Figure 1. Bacteriophage-mediated induction of TNFα expression in HT-29 epithelial cells.

**

**Supplementary Figure 1. Bacteriophage-mediated TNFα expression.** TNFα expression was determined in primed HT-29 cells stimulated with 10^3^ PFU/HT-29 bacteriophage (A.) T4, (B.) ϕX174, and (C.) M13 for 6 hours. Respective controls for each experiment were LPS, bacteriophage, and a combination of bacteriophage with LPS. The comparative 2^-ΔΔCt^ method was used to quantify gene expression level changes in the respective controls relative to SM buffer after normalization to the housekeeping gene GAPDH. All graphs are representative of n ≥ 3 experiments and depict mean with SEM of n ≥ 3 replicates from an individual experiment: each dot indicates the replicate value. Analysis: one-way ANOVA with Tukey’s test for multiple comparisons. ****** = P < 0.01, ******* = P < 0.001, **ns** = not significant.

**Supplementary Figure 2. Impact of filamentous bacteriophage on viability and IL-8 expression in non-primed HT-29 cells.**





**Supplementary Figure 2. Impact of filamentous bacteriophage on viability and IL-8 expression in non-primed HT-29 cells.** (A.) Viability of HT-29 in response to stimulation with M13 (10^3^ PFU/HT-29) and Fd (10^3^ PFU/HT-29) at 24 hours. Additional controls are SM buffer, LPS (0.5 ng/mL), a combination of M13 (10^3^ PFU/HT-29) and LPS (0.5 ng/mL), a combination of Fd (10^3^ PFU/HT-29) and LPS (0.7 ng/mL), and DMSO (20%) (B.) IL-8 expression measured in non-primed HT-29 cells stimulated with M13 (10^3^ PFU/HT-29) for 24 hours. Additional controls are SM buffer, LPS (0.5 ng/mL), a combination of M13 (10^3^ PFU/HT-29) and LPS (0.5 ng/mL) (C.) IL-8 expression was measured in non-primed HT-29 cells stimulated with Fd (10^3^ PFU/mL) for 24 hours. Additional controls are SM buffer, LPS (0.71 ng/mL), a combination of Fd (10^3^ PFU/HT-29), and LPS (0.71 ng/mL). The comparative 2^-ΔΔCt^ method was used to quantify gene expression level changes relative to SM buffer after normalization to the housekeeping gene GAPDH. All graphs are representative of n ≥ 3 experiments and depict the mean with ±SEM of n ≥ 3 replicates from an individual experiment, each dot indicating the experiment value. Analysis: one-way ANOVA with Tukey’s test for multiple comparisons. ******* = P < 0.001; ******** = P < 0.0001.

**Supplementary Figure 3. Filamentous bacteriophage-mediated dose-dependent IL-8 expression.**

**

**

**Supplementary Figure 3. Dose-dependent IL-8 expression.** in HT-29 cells in response to bacteriophages (A.) Fd and (B.) M13 at 24 hours. Concentrations were designated as a ratio of bacteriophages to HT-29 cells, and the ratios that were used ranged from 10^-1^-10^4^ bacteriophage PFU/HT-29. The comparative 2^-ΔΔCt^ method was used to quantify gene expression level changes relative to SM buffer after normalization to the housekeeping gene GAPDH. All graphs are representative of n ≥ 3 experiments and depict mean with SEM of n ≥ 3 replicates from an individual experiment: each dot indicating the replicate value. Analysis: one-way ANOVA with Dunnett’s test for multiple comparisons. ****** = P < 0.01, ******* = P < 0.001.

**Supplementary Figure 4. Filamentous bacteriophage** **mediated dose-dependent IL-8 expression in HT-29 cells.**

**

**

**Supplementary Figure 4. HT-29 cell bacterial internalization is affected by M13.** (A.) Confluent HT-29 cells were co-incubated with 10^7^ CFU/mL *E. coli* strain W1485 or M13 (10^7^ PFU/mL) and *E. coli* concurrently (phage-bacteria ratio or MOI =1) for 6 hours prior to determining bacterial internalization. (B.) HT-29 cells were stimulated with different concentrations of M13 (10^0^-10^3^ PFU/HT-29) for 6 hours prior to being infected by 10^7^ PFU/mL *E. coli* W1485 for 6 hours. All graphs are representative of n = 3 experiments and depict the mean with ±SEM of n = 9 replicates; each dot indicates the replicate value. Statistical analysis was computed based on the 9 technical replicates.; each dot indicating the replicate value. Analysis: (A.) Two-tailed Student’s t-test; (B.) one-way ANOVA with Dunnett’s test for multiple comparisons. ****** = P < 0.01, ******* = P < 0.001.
